# A qualitative analysis of the effect of a community-based primary health care programme on reproductive preferences and contraceptive use among the Kassena-Nankana of northern Ghana

**DOI:** 10.1186/s12913-016-1325-6

**Published:** 2016-03-05

**Authors:** Maxwell Ayindenaba Dalaba, Allison E. Stone, Abigail R. Krumholz, Abraham R. Oduro, James F. Phillips, Philip B. Adongo

**Affiliations:** Navrongo Health Research Centre, P.O.Box 114, Navrongo, Upper East Region Ghana; Columbia University Mailman School of Public Health, Heilbrunn Department of Population and Family Health, 60 Haven Ave. B-2, New York, NY 10032 USA; Navrongo Health Research Centre, P.O.Box 114, Navrongo, Upper East Region Ghana; Department of Social and Behavioral Science, School of Public Health, University of Ghana, PO Box LG 14, Legon, Ghana

**Keywords:** Community-based health planning and services, Contraceptive knowledge, Gender, Male involvement, Reproductive preferences, Ghana

## Abstract

**Background:**

In 2000, Ghana launched the Community-based Health Planning and Services (CHPS) initiative to improve access to health and family planning services. This initiative was based in part on research, known as the Navrongo Project, conducted in the Kassena-Nankana district (KND) between 1994 to 2003 which demonstrated significant impact on fertility and child mortality. This paper examines current contraceptive perceptions in communities that were exposed to the Project’s service models over the 1994 to 2003 period, and the post-experimental policies of the CHPS era.

**Methods:**

Qualitative study was conducted in the KND of Ghana from June to September, 2012, by convening 8 male and 8 female FGD panels as well as 8 in-depth interviews of community leaders. Data collection was stratified by original experimental cell of the Navrongo Project to permit appraisal of social effects of contrasting experimental conditions. Inductive content analysis was performed with QSR Nvivo 10 to identify predominant themes.

**Results:**

While findings show that exposure to community-based services was associated with enhanced approval of birth spacing and limitation, this view is grounded in perceptions that childhood survival has improved. Nonetheless, concerns were expressed about contraceptive side effects, prominently permanent sterility. Strategies for male outreach and community engagement originally introduced during the Navrongo Project have not been sustained with CHPS scale-up. The apparent atrophy of attention to the needs of men may explain the resistance of some males to the notion of female reproductive autonomy and the practice of some women to adopt contraception in secret. Despite this apparent programmatic dearth of male engagement, there is evidence to suggest that social impact of the original male engagement strategy persists in communities where male mobilization was combined with doorstep provision of family planning care during the Navrongo Project.

**Conclusion:**

Community-based services fostered attitudinal change towards family planning in a traditional sub-Saharan African setting. Sustained exposure to primary health care that have improved the survival of children has made the use of contraception more acceptable. Efforts should be embedded in primary health care programmes that address concerns about child survival while also consigning sustained priority to the information needs of men.

**Electronic supplementary material:**

The online version of this article (doi:10.1186/s12913-016-1325-6) contains supplementary material, which is available to authorized users.

## Background

Ghana ranks among the earliest countries in Africa to adopt policies that aim to improve access to family planning services [1]. Yet, despite these longstanding commitments, contraceptive use remains low, particularly in northern regions of the country where poverty is most pervasive [2]. In response to evidence that accessible care was needed, the government adopted a model for community-based service delivery known as the Community-Based Health Planning and Services (CHPS) Initiative in 2000. The CHPS model was based, in part, on research conducted in the Kassena-Nankana district between 1994 and 2003 which demonstrated that community-based primary health care could have a pronounced impact on fertility and child mortality [3, 4]. This study, known as the “Navrongo Project”, was a quasi-experimental test of the hypothesis that, introducing health and family planning services in a traditional African societal setting would introduce reproductive change and improve childhood survival.

The Navrongo Project was configured with two experimental arms, one involving the posting of trained and paid nurses, known as Community Health Officers (CHOs), and the other involving volunteers and community mobilization activities termed “the *zurugelu* approach” [5]. Prior to the Project, nearly all health workers were limited to clinical duties owing to revenue and organizational constraints that prevented the development of health posts and strategies that would enable these nurses to live and work directly in village locations. The project developed community outreach strategies for developing facilities and support for nurses, who were provided with training in doorstep provision of health services and given the new CHO designation. The zurugelu (meaning “togetherness” in the local dialect) arm involved community-engagement activities designed to build male leadership, communication and participation in reproductive health services, and to expand women’s participation in community forums that had traditionally been the purview of men [4].

Since the two experimental arms could be implemented independently, jointly, or not at all, communities involved in the Navrongo Project comprised a four-celled plausibility trial. Cell 1 tested only the zurugelu approach, Cell 2 tested only the CHO approach, Cell 3, the combined cell, tested the joint-implementation of the zurugelu and CHO approach, and Cell 4 served as a comparison area where service provision was located in sub-district facilities.

At the end of the study, the fertility impact of the Project was significant, but limited to Cell 3 alone [6]. The fertility rate was 15 % below comparison area levels, corresponding to one birth less per woman. Childhood mortality rates were reduced by over half in three years in both Cells 2 and 3 [7].

Due to this dramatic success, in 1999 the Ministry of Health and Ghana Health Service (GHS) officially adopted the Cell 3 approach of the Navrongo Project as the model for community-based primary health care. The Community-Based Health Planning and Services (CHPS) Initiative was launched in 2000 as a national programme for scaling-up this policy [1].

One key component of the CHPS initiative is the effective provision of family planning information and services, including doorstep provision of oral, injectable and barrier contraceptive and referral for intrauterine devices and other long-acting methods [4]. CHPS health workers educate community members on the benefits of family planning with the goal of changing perceptions and attitudes and increasing contraceptive uptake. However, introducing and sustaining reproductive change requires attention to the needs and concerns of both men and women so that the social determinants of unmet need for contraception can be addressed by programme action and support. Analyses of long term fertility trends in the Navrongo Project area have revealed that the pace of reproductive change has stalled in communities where fertility effects were originally established [8]. This suggests that the scale-up of CHPS services has not proceeded in a manner that sustains the fertility reduction achieved by the model during the Navrongo Project. This paper reports community perceptions on activities during the Navrongo Project and the current CHPS era to determine reasons why the fertility impact stalled during scale-up. We also examined the extent to which the CHPS programme has changed social and behavioural attitudes and gender customs towards the use of family planning.

This paper is part of a larger study that aimed to assess the impact of the Navrongo “Zurugelu Appraoch” on men’s concerns about family planning and reproductive health services.

## Methods

### Study site

The study was carried out in the Kassena-Nankana East and West Districts of Ghana’s Upper East Region. Until recently, these comprised a single district known as Kassena-Nankana District (KND). The combined KND area has a population of 152,000 people and covers approximately 1675 km^2^ [9]. The area has a dry, hot season from October to April, with a rainy season from May to September. Subsistence agriculture is the main occupation. There are two main ethnic groups in the districts: the Kassena comprising about 49 % of the population and the Nankana comprising about 46 % of the population. The remaining minority is comprised mainly of Buli. Although the Kassena and Nankana languages differ, their cultural norms and social institutions are similar. KND has a district hospital located in Navrongo town, the capital of the Kassena-Nankana East District, which serves as a referral point for six health centers, one private clinic and 27 CHPS community health compounds in the two districts. These CHPS compounds have resident CHOs offering doorstep services to community members [1, 4]. There are two community clinics jointly run by the Catholic Diocesan Development Office and the District Health Administration that provide services to the communities.

The Navrongo Health Research Centre operates a Health and Demographic Surveillance System (HDSS) which continuously monitors demographic events, household composition, and other information about health services and health status for the entire KND population, as well as relative household economic status and educational attainment [9].

### Study design and data collection

Qualitative data for this study were collected between June and September 2012 by convening focus group discussions (FGDs) and in-depth interviews (IDIs). Experienced graduate-level research officers conducted the FGDs and IDIs in relevant local languages. Prior to data collection, interviewers were provided with a week of training on the research protocol and interview procedures.

A total of eight male and eight female FGD panels were convened, as well as eight IDIs with community chiefs and elders. Community respondents were proportionally drawn from the original experimental cells of the Navrongo Project to allow for a comparison of current community knowledge and acceptance of family planning across communities with contrasting degrees of exposure to community-based primary health care. FGD participants were stratified according to time period, with half of the FGDs comprised of individuals who were familiar with the original Project and therefore potentially able to reflect on both past and present circumstances of reproductive health services and attitudes. The other half of FGD respondents were younger and therefore only familiar with the current CHPS programme and current attitudes. Thus, out of the 16 FGDs conducted, 4 were with older men, 4 with older women, 4 with younger men and 4 with younger women (Table 1).

**Table 1:** Description of respondents

	Respondents	Exposure	Type of interviews	Number of interviews
	FGDs (16)			
1	Older men –35 years and above	CHFP and CHPS	FGD	4
2	Younger men – less than 35 years	CHPS	FGD	4
3	Older women –35 years and above	CHFP and CHPS	FGD	4
4	Younger women – less than 35 years	CHPS	FGD	4
	IDIs (8)			
5	Chiefs	CHFP and CHPS	IDI	2
6	Chiefs	CHPS	IDI	2
7	Elders	CHFP and CHPS	IDI	2
8	Elders	CHPS	IDI	2

Discussion guides were used for all IDIs and FGDs and covered issues such as current activities related to the provision of family planning, perceptions of the importance of these services, and perceptions of social or reproductive change over the past 15 years that may have affected family planning demand or service supply. Interview guides (FGD and IDI) are presented as Additional file 1. All IDIs and FGDs were conducted in one of the local languages (Kassem or Nankam), audio-recorded and subsequently transcribed into English. All transcriptions were reviewed and inductive content analysis was performed using QSR Nvivo 10 software to identify broad and sub-themes. A codebook was developed based on emerged themes and two independent researchers coded the transcripts.

### Ethical considerations

Community entry activities included meetings with chiefs and elders in all study communities before commencement of data collection. The study protocol was reviewed and approved by the Navrongo Health Research Center and Columbia University Institutional Review Boards. Oral informed consent was obtained from all participants in the FGDs and IDIs.

## Results

### Programmatic exposures

The research team was prepared to observe differences in community perceptions and attitudes when comparing the responses of older FGD participants familiar with both the Navrongo Project and the current CHPS programme to younger respondents familiar with only the CHPS programme. However in the analysis of the data variations were not observed in the themes arising from the two different groups. Instead, predominant themes identified were similar regardless of programmatic exposure. While the two programe are distinct from a practitioner’s perspective, the differences between the Navrongo Project and CHPS programme seem to be less apparent from the perspective of community members. Because of this finding, we have presented the results according to shared themes rather than separating them according to programmatic exposure.

### Awareness of family planning methods

Awareness of family planning services among community members in the study communities was virtually universal. When respondents were asked to mention types of contraceptives, most were able to mention several methods, differentiating them by the time period during which the method can prevent pregnancy. The one-month injection, three-month injection, five-year implant, sterilization, daily tablets and condoms were among the types of family planning methods mentioned by community members:

*“What I know is that we have the three months methods that you can use. I have also heard that there is five years method as well.” (Man, exposed to Navrongo Project)*

*“they are really many. There are some injections for three months; others are pills and many others. If you try one method and it does not work, you are advised to try another.” (Woman, exposed to CHPS only).*

The most frequently cited and discussed method was the three-month injection, Depot-Medroxy Progesterone Acetate (DMPA), although some respondents mentioned that stock shortages of this method were common. As stated by one community member:

*“What I will suggest is that the devices should always be made available because mostly at the CHPS compounds the women patronize the Depo but sometimes they run short of it so they should make the devices available all the time, and then for the sake of those who cannot afford the user fees if they can add it into the health insurance it will benefit those people.” (Woman, exposed to CHPS only)*

Some methods requested by respondents are only provided at hospitals, especially the five year sub-dermal methods that were not available during the Navrongo Project era. Although these methods are available to clientele referred to a hospital, the distance or lack of accessibility was cited as a source of frustration to couples.

### Perceived benefits of family planning

The majority of men and women expressed positive views about family planning and stated that there is greater acceptance of family planning among community members now than in the past. This acceptance was attributed to the CHPS programme and the subsequent realization of the benefits of family planning, as described by one community member:

*“First when FP was introduced to community members, the number using FP was low, but the number of people who do FP has now increased and men even take their wives to the health centres to do FP. That is the change in the FP aspect and it is as a result of the CHO activities.” (Elder, exposed to Navrongo Project and CHPS)*

The majority of respondents mentioned that family planning helps individuals in birth spacing and limiting the number of children they have, citing benefits for the health of children as well as mothers. Also many respondents noted the reduced mortality of children in recent years, due in part to the childhood immunizations and curative services provided by CHPS, as a contributing factor in the increased acceptability of family planning. One older woman described this as follows:

*“I think FP is good because the rate at which children die these days is reduced. This is because the nurses give children medicine against diseases and this has really helped us a lot. This is why we have to accept FP too.” (Woman, exposed to Navrongo Project and CHPS).*

Some respondents said that, family planning also promotes harmony and fidelity between couples, noting that, without family planning, some women attempt to avoid pregnancy by denying their husbands sexual relations, which can lead to spousal discord and intimate partner violence. As an elder in the community noted:

“*They have seen the benefits of it and the fact that it helps you to space your children the way you want it. This is one reason that has made them to understand and agree to practice it. If you don’t do family planning, you may have problems with your husband because of sex. This used to happen in the past but because of family planning, couples don’t quarrel again because anytime the man wants sex the woman will give him knowing very well that she will not become pregnant in the process. So family planning has come to separate quarrel between couples for us.” (Elder, exposed to Navrongo Project and CHPS)*

Community members said that their sources of information about family planning include health workers, health volunteers and traditional leaders. Two chiefs described their roles with regards to community attitudes towards family planning as follows:

*“We have talked to them and they have agreed and there are changes. It is no longer like it used to be. Now you can see a woman who has given birth and that child can take care of the sibling (birth spacing). So for that it is helpful. If you give birth to children with no spacing and you cannot take care of them and when you see them you think they are twins that is not good*.” *(Chief, exposed to Navrongo Project and CHPS).*

*“In the past when the family planning was not there, children were like property and if you had many children it would show who you were. Because of that people at that time preferred to have many children. Now because of the family planning campaign all over, people have understood it and preferred to have fewer children.” (Chief, exposed to CHPS)*

Despite this evidence of a favorable climate of opinion about family planning, gender differences are evident. Respondents claimed that women have greater demand for family planning compared with men. Secrecy about family planning practice is common among women, as husbands are sometimes opposed to family planning. The following quotes illustrate varying opinions on surreptitious use of family planning by women:

*“Now things have changed and even if the man has not agreed for me to do the family planning, I have to go and do it without his notice because when I give birth to so many children, it is me that will suffer with the children and not the man. I will even agree for the man to beat me than saying that I will not do it. The family planning has really helped we the women a lot and we can now give birth to fewer children that we can take good care of them.” (Woman, exposed CHPS).*

“*When the woman goes to do [family planning] before telling the man, it is not good.” (Man, exposed to CHPS).*

*“..there was a day I went to a nurse and explained to her the reason why I want to do family planning. So she did it for me and said I should take the book home but I said no, she should keep the book there for me. When I got home my husband asked [where I went] and I said I went to my friend.” (Woman, exposed CHPS)*

### Misconceptions or side effects of family planning

Notwithstanding the benefits of family planning and considerable support for it among community members, some respondents expressed misconceptions or anxieties about family planning. Some women were concerned that infertility can result from contraceptive use. The following responses highlighted some anxieties:

*“The other reason why some men do not want their wives to do family planning is that if you give birth to only one child and you do family planning, a time will come whereby you want to become pregnant and it may not be possible because of the family planning you did.” (Woman, exposed to CHPS)*

*“Yes some women if you are talking to them about family planning they tell you if they do family planning they cannot deliver again because some do it and when they stop using it then they bleed a lot so because of that they prefer to stay without using family planning.”(Woman, exposed to CHPS).*

Several respondents also mentioned weight gain as an undesirable side effect of family planning use.

*“The problem about the family planning is the weight gain. Sometimes, when you take the injectable contraceptives, like the 3 months or the 5 years one, you put on weight a lot. That is the problem, so if they can check this, it will help us a lot” (Woman, exposed to CHPS).*

*“The 5 years family planning would have been the best method. However, when you take the 5 years injection, you gain a lot of weight, and everybody will know that you are on family planning. This sometimes spoil your beauty” (Woman, exposed to Navrongo Project and CHPS)*.

### Family size

The majority of respondents mentioned that in the past couples would seek to give birth to as many children as possible, as they were regarded as a form of wealth. Also, because childhood mortality was high, having many children was a form of insurance that some children would survive childhood. Respondents noted that at present, with the greater reach of child health services through CHPS and their success in reducing child mortality, in conjunction with current economic difficulties, it is better to have fewer children so that parents can properly provide for them. The preferred family size mentioned by respondents was nonetheless broad, ranging between two and six children. For example:

“*If she gives birth to five it is okay because they used to say that if you don’t give birth to many children and there is outbreak of measles, CSM, and they die, how many will be left, but these days we don’t hear about measles, we don’t hear about CSM, so if you give birth to five it is enough.” (Elder, exposed to Navrongo Project and CHPS)*

*“People in this community are now wise following the health education the people get. they now give birth to four or five and stop.” (Man, exposed to Navrongo Project and CHPS).*

Nonetheless, a few men and women still believe in having a large family size, with as many children as God provides:

*“Hahahaha (laughter), it is the number that God wants you to give birth to.” (Woman, exposed to CHPS)*

*“In my opinion, I will say ten children, 5 boys and 5 girls, that is what we think.” (Man, exposed to CHPS)*

### Sex preference

On the issue of the preferred sex of children, a majority of the respondents expressed the view that sex of children does not matter, with the explanation that it is God’s will. As a chief noted:

*“For me, it is God who knows best because every child is a child and so any child God gives you, will you refuse? All children are children and so when God give you whether boys or girls, they are all children.” (Chief, expose to Navrongo Project and CHPS)*

However, a few respondents insisted that having children of both sexes is important because daughters bring wealth to the family through marriage while sons inherit their father’s wealth and sustain the lineage. Accordingly, couples said they would continue bearing children until their desired gender mix is attained:

*“Well gender matters, it is so crucial and if there is a balance in sex (boys and girls), that’s perfect. But the worry is,who inherits the father after he is gone. The issue of only girls is our problem but where too you have only boys but no girls the parents too worry. It is a worry in the sense that should there no be girls how will the world look like? The issue is to keep your family going and our culture does not allow a girl to stay in the house to take over the house. So if couples have only girls, they will have to keep delivering because they will need a boy to add.” (Elder, exposed to CHPS).*

## Discussion

This study explored the impact of the Navrongo Project and the subsequent roll-out of the CHPS programme on community perceptions of family planning, with a particular focus on the possibility that programme activities have had social impact. Responses show that knowledge of family planning was prevalent among all respondents and that the benefits of contraception were widely acknowledged. Moreover, actual use of modern contraceptives was perceived to be more widespread in the communities now than 15 years ago at the outset of the Navrongo Project. Responses provide evidence of a general change in attitudes from the days of the Project, during which community members often believed the use of contraceptives by women would lead to rejection, disagreement, and conflict among family members [10]. Shifts in the acceptability of family planning were grounded in the perception that mortality of children has declined, to a large extent because childhood immunizations and health services have improved survival.

Nonetheless, there were misconceptions and anxieties raised by respondents that concern the safety of contraceptive use in all communities. In particular, respondents often believed that methods caused women to become infertile or could impair fecundity. Dramatic weight gain was also mentioned as an undesirable side effect of family planning. These findings are consistent with other studies that have reported fears of side effects as a primary reason why women choose not to use contraceptives [11, 12]. Clearly, such concerns merit careful attention to ensure that CHOs are trained to provide accurate and comprehensive community education about the potential side effects of short and long term contraceptive methods that counters false rumours and beliefs.

However, the most prominent of the concerns about family planning came from male respondents who expressed discomfort with the possibility of female reproductive autonomy. Concerns of men that have been noted in the past [13] persist despite two decades of service activities in study localities. Given that one of the main strategies in the success of the Navrongo Project was effective male involvement in family planning activities to mitigate social costs that women confront in using contraception, there is an evident need to strengthen this component of the current CHPS programme and to encourage all community members to join the effort to promote the use of family planning. This observation is not unique to the Kassena-Nankana of northern Ghana [2]. Despite the fact that profound social and economic factors distinguish the cultures of northern Ghana from the ethno-linguistic groups of southern Ghana, our results provide evidence of remarkable similarity in the views and concerns of men to similar studies that have been conducted elsewhere in Ghana [14, 15]. The need to engage men in groups to discuss the role of wisdom and manhood in protecting women’s rights was demonstrated as an effective strategy of the Navrongo Project that has not been prominently replicated by its scale-up as CHPS [8, 16].

Various themes about family planning, imparted by CHPS supervisors to groups of men, could address their worries about their status and reproductive control by placing discussion about family planning services in the open so that men have a forum to express their views. These simple, low-cost, and effective strategies of the Navrongo Project should now be replicated by CHPS on a large scale [17].

The preference for large family size, a predominant attitude in the past, appears to have changed substantially throughout the study area, with the value and benefits of having a smaller family becoming pivotal in family planning acceptance. In the past, there was a strong preference for large families and for many sons in the study area [18–20]. However, our study revealed changes in this preference as a majority of respondents believed that the sex of a child is God’s making and it does not matter whether a child is male or female. Notwithstanding that, some respondents still insisted that sex is important and that having children of both sexes is ideal. In polygynous northern Ghana, men may try to continue child bearing with their spouse or another partner until their desired mix of sexes of children is attained. As family planning use becomes more prevalent, it is possible that the desire for a mix of sexes will influence contraceptive use dynamics. Whereas couples traditionally preferred sons to daughters, fertility transitions may be slowed by emerging preferences for a mix of sexes among children. With these changes in sex preference, individuals have come to the realization that uncontrolled fertility may not only lead to poverty in the family but also has important effects on maternal and child health.

## Conclusion

Ghana’s Community-based Health Planning and Services (CHPS) Initiative is aimed at improving access to comprehensive primary health and family planning services. The initiative has contributed in a change of attitudes toward contraception as more individuals in traditional societies approve of the use of family planning services. Nonetheless, concerns and worries that arise from gender stratification customs remain prominent; factors that underlie traditional high fertility norms and reproductive preferences persist. For this reason, demand for family planning remains fragile, and the requirements of a robust and culturally sensitive programme are evident. Efforts are still needed to address misinformation about the side effects of contraceptive use, while also ensuring that accurate and complete information is freely available and well understood. Balanced and thoughtful communication to address community misconceptions about family planning is essential to the effective implementation of the programme. Additionally, to further improve and sustain the use of family planning services, programmes targeting males for acceptance should be strengthened with outreach to groups of men via engagement with their social networks and traditional communication systems. In a setting like rural northern Ghana where educational attainment is low, achieving community understanding of the value of contraceptives is an essential but gradual process, requiring sustained community engagement about the benefits of family planning.

## Additional file

Additional file 1: Interview guides. (DOC 43 kb)

## Abbreviations

CHPS: community-based health planning and services
CHFP: community health and family planning
CHO: community health officer
GHS: ghana health services
IUD: intrauterine device
KND: kassena-nankana district
FGD: focus group discussion
IDI: in-depth interviews
HDSS: health and demographic surveillance system
DMPA: depot-medroxy progesterone acetate
FP: family planning
