# Supplementary material for: A qualitative analysis of the effect of a community-based primary health care programme on reproductive preferences and contraceptive use among the Kassena-Nankana of northern Ghana
Source: BMC Health Serv Res. 2016 Mar 5;16:80. doi: 10.1186/s12913-016-1325-6 (PMC4779239; doi:10.1186/s12913-016-1325-6)
Supplement: Additional file 1: — Interview guides. (DOC 43 kb) [file 12913_2016_1325_MOESM1_ESM.doc]

**INTERVIEW GUIDES**

1. **Focus Group Discussion Guide**

**Title: The effect of a community-based primary health care programme on reproductive preferences and contraceptive use among the Kassena-Nankana of northern Ghana**

**INTRODUCTION:**

Good morning/afternoon. We are pleased to have you here to discuss health and other related issues. We are from the Navrongo Health Research Centre (VAST). Many of you already know each other well, but why don’t we begin with everyone saying their names.

**CHPS Services**

1. What is the role of the community health officers in your community?

- What do you think are qualities of effective CHOs?

2. Do you think the nurses here are well-engaged with the community?

- Does the community support their work?
- If so, in what ways?

3. Has the work of the CHOs changed from when they were first introduced to your community?

- If so, how?

4. What is the role of health volunteers?

- What are good qualities for volunteers to have?
- Do you think this community has effective health volunteers?

5. Has the role of health volunteers changed from when they were first introduced here?

- If so, how?

**Family Planning**

6. In your community, how many children do families usually want to have?

7. Do families prefer to have boys or girls, or does it not matter?

8. Do husbands and wives usually agree on how many children to have?

- What happens when husbands and wives disagree about how many children they want to have?

9. What do members of your community think about family planning?

- Do you think that women in the community are interested in using family planning?
- Do men in this community support the use of family planning?

10. Are current issues about family planning different than they were when you were young? If so, how are they different?

- What did men think about family planning in the past?
- What did women think about family planning in the past?
- What do you think led to the change in how people think about family planning?

11. When you were young, did people in your community use family planning?

- Has there been any change since then?
- If so, what has caused this change?

12. Currently, what types of family planning methods are available in your community?

13. In your opinion, is it important that family planning services are available in your community?

- Do you think it would be good or bad if more people used family planning?

14. What role should nurses play regarding the use of family planning?

- Should they promote family planning?
- Do you think that they are more or less involved in promoting family planning than in the past?

15. What role should health volunteers play in regards to family planning?

- Has the role of volunteers in family planning changed over time?
- If so, how?

**Barriers / User Fees**

16. Is there anything that prevents people from using family planning if they want to?

- (Probe: spousal preference; religious beliefs; social pressure)

17. Can you think of anything that would increase the use of family planning in your community?

18. Do you think user fees have an impact on how many women use family planning?

- If so, what is the impact?
- Who normally pays the user fees when a woman uses family planning?
- Do some women do not use family planning because they cannot ask their husbands for money for the user fees?

19. Thank you so much for your time. Is there anything else you’d like to add that we have not already discussed?

1. **In-Depth Interviews with Chiefs and Elders**

**Title: The effect of a community-based primary health care programme on reproductive preferences and contraceptive use among the Kassena-Nankana of northern Ghana**

**INTRODUCTION:**

Good morning / afternoon Sir. My name is ______________ and I am from the Navrongo Health Research Centre. I would like to ask you some questions about the health of your community and the health services provided in this area.

**Background and CHPS Services**

1. When did you become the Chief/Elder of your community?

2. How would you describe the health of your community?

- What about the health of children in the community?
- How would you compare the health of children today as compared to ten years ago?

3. Have you noticed any changes in the community in terms of child deaths?

- If so, what would you say accounts for these changes?

4. In general, how does your community learn about health practices?

5. Are you involved in health activities in your community?

- If so, in what way are you involved?

6. Do you remember when nurses were first introduced to your community?

- If so, can you describe the process by which they were introduced to your community?
- Was the community involved in this process?
- Were you involved in this process?
- How were nurses integrated into the community?

7. Do you remember when health volunteers were first introduced to your community?

- If so, can you describe the process by which they were introduced to your community?
- How were the volunteers selected?
- Was the community involved in the process of selecting and deploying the volunteers?
- Were you involved in this process?
- How were volunteers integrated into the community?
- Were nurses and health volunteers introduced to your community at the same time, or did one come before the other?

8. What is the role of the nurses in your community?

- What are their main activities?
- What do you think are qualities effective nurses?

9. Do you think the nurses here are well-engaged with the community?

- Does the community support their work?
- If so, in what ways?

10. Has the work of the nurses changed from when they were first introduced to your community?

- If so, how?

11. What is the role of health volunteers?

- What are their main activities?
- What are good qualities for volunteers to have?
- Do you think this community has effective health volunteers?

12. Has the role of health volunteers changed from when they were first introduced here?

- If so, how?

13. Are community durbars about health topics held in your community?

- If so, how frequently?
- What are their key messages?

**Reproductive health and Family Planning**

14. In your opinion, what is the ideal number of children a woman should have in her lifetime?

15. Is it better for a woman to have boys or girls, or a combination of the two? Or does gender of children not matter?

16. Do husbands and wives usually agree on how many children to have?

- What happens when husbands and wives disagree about how many children they want to have?
- What happens if a man and a woman have been married for some time but the marriage does not produce any children?

17. Would you say that members of your community want more or fewer children compared to 15 years ago?

- If there have been some changes, what do you think caused the changes?
- (Probe: Influence of religion, cost of living, education and marriage)

18. What are your opinions about family planning?

- Do you think that family planning has a positive or negative effect on your community?
- Why?

19. Have your views about family planning changed at all over time?

- If so, how have they changed?
- What caused the change in your views?

20. What do members of your community think about family planning?

- Are women in the community interested in using family planning?
- Do men in this community support the use of family planning?

21. Are current opinions about family planning different in recent times than they were fifteen years ago? If so, how are they different?

- What did men think about family planning fifteen years ago?
- What did women think about family planning fifteen years ago?
- What do you think led to the change in how people think about family planning?

22. Did people in your community use family planning fifteen years ago?

- Has there been any change since then?
- If so, what has caused this change?

23. How do people in your community learn about family planning?

- (Probe: home-to-home visitation, health talks at the clinic, health durbars)

24. In your opinion, is it important that family planning services are available in your community?

- Do you think it would be good or bad if more people used family planning?

25. Do you have a role with regards to community attitudes towards family planning?

- If so, what is your role?

26. What role should health workers play regarding the use of family planning?

- Should they promote family planning?
- Do you think health workers have an impact on community attitudes about family planning?
- Do you think that the current health workers are more or less involved in promoting family planning than in the past?

27. What role should health volunteers play in regards to family planning?

- Do you think volunteers have an impact on community attitudes about family planning?
- Has the role of volunteers in family planning changed over time?
- If so, how?

**Barriers / User Fees**

28. Is there anything that prevents people from using family planning if they want to?

- (Probe: spousal preference; religious beliefs; social pressure)

29. Can you think of anything that would increase the use of family planning in your community?

30. Do you think user fees have an impact on how many women use family planning?

- If so, what is the impact?
- Who normally pays the user **fees** when a woman uses family planning?
- Do some women not use family planning because they cannot ask their husbands for money for the user fees?

31. Thank you so much for your time. Is there anything else you’d like to add that we have not discussed already?
